# Commentary: Imaging Biomarkers and Pathobiological Profiling in a Rat Model of Drug-Induced Interstitial Lung Disease (DIILD) Induced by Bleomycin

**DOI:** 10.3389/fphys.2021.691650

**Published:** 2021-07-28

**Authors:** Sotirios I. Sinis, Sotirios G. Zarogiannis

**Affiliations:** ^1^Department of Physiology, Faculty of Medicine, School of Health Sciences, University of Thessaly, Larissa, Greece; ^2^Department of Respiratory Medicine, Faculty of Medicine, School of Health Sciences, University of Thessaly, Larissa, Greece

**Keywords:** animal models, bleomycin, imaging biomarkers, translational research, drug induced interstitial lung disease

Drug-induced interstitial lung disease (DIILD) is a clinical entity bearing significant morbidity and mortality that presents with non-specific symptoms, more frequently cough, dyspnea, wheezing, hemoptysis, fever, and pleuritis (Matsuno, 2012; Carrington et al., 2018). Management heavily relies upon early suspicion, elimination of other causes and withdrawal of the offending agent. High-resolution computed tomography (HRCT) is an integral part of the diagnostic algorithm for DIILD albeit it does not provide pathognomonic evidence in most cases. Pulmonary insult from bleomycin, a chemotherapeutic used in Hodgkin lymphoma and germ cell tumors, is a commonly encountered DIILD paradigm characterized by specific radiological patterns (Skeoch et al., 2018). Series of case reports involving patients undergoing chemotherapy offer insight regarding the potential usefulness of HRCT with FDG-PET in the management of DIILD. During follow-up for assessment of treatment outcomes, new areas of FDG uptake in the lung parenchyma should alert the clinician as they likely represent early stages of an inflammatory process prior to the appearance of symptoms or notably HRCT abnormalities (Paschali et al., 2017; Beyhan Sagmen et al., 2019). On the other hand, pulmonary lesions showing signal attenuation could represent resolution of the disease or progression to fibrosis (Taywade et al., 2016; Paschali et al., 2017). There are many unanswered questions pertaining to the onset and temporal progression of DIILD that would be ethically, logistically and financially unsound to resolve in human studies. On these grounds, preclinical animal models that mimic the disease development of DIILD should be standardized in order to generate extrapolatable data on imaging biomarkers, a promising tool that will pave the way for advancing timely diagnosis and more effective therapy (Mahmutovic Persson et al., 2020b).

A great start toward this direction was achieved in a recently published study in *Frontiers in Physiology* by Mahmutovic-Persson et al. establishing a rat model of DIILD induced by bleomycin, with a very elaborate set of data comprising temporal evaluation of imaging biomarkers (MRI and PET/CT), lung tissue gene expression patterns, BALF cell counts and cytokine profiles in the time course of up to 28 days post bleomycin administration (Mahmutovic Persson et al., 2020a). This is one of the first comprehensive studies employing the conventional bleomycin-lung injury model, which is praised for its clinical relevance, reproducibility and repeatability, in the context of preclinical model development for DIILD surveillance through imaging biomarkers. Prior to this work, literature involving intratracheal instillation of bleomycin mainly comprised preclinical fibrosis and IPF models which take advantage of the latent stage of the response starting from day 14 with maximal intensity at days 21–28 to recapitulate and study fibrosis in humans. Considerable heterogeneity in experimental conditions often permits qualitative comparison of datasets. Gadolinium and gallium based probes in conjunction with MRI or PET have been successfully tested in tracking fibrosis progression and resolution (Caravan et al., 2013; Chen et al., 2017; Desogere et al., 2017). On the other hand, a limited set of evidence describes employment of labeled polymorphonuclear cells and leukocytes that visualize the preceding inflammation that culminates at approximately day 7 post exposure (Das et al., 1988; Bondue et al., 2015). Another novel aspect of the study of Mahmutovic-Persson et al. is the use of MRI and PET post-bleomycin administration (1,000 iU once) to stratify rats based on lung volume expansion in High and Low responders (Mahmutovic Persson et al., 2020a). A quantitatively similar volume expansion assessed by MRI has been previously reported by Egger et al. in Sprague-Dawley rats receiving 2 and 3 mg/kg of bleomycin by oropharyngeal aspiration (Egger et al., 2014). MRI experiments showed that the increased lung volume in high-responders persisted and fibrotic lesions regressed at day 28, in contrast to the low responders whose MRI imaging biomarkers returned to baseline. Uptake pattern of FDG peaked in conjunction with inflammation at day 7 in exposed rats but remained elevated longer in the high responders (14 days) and was able to discriminate the relapse at day 28. This corroborates animal and patient derived data suggesting FDG could also be a biomarker of fibrosis (Bondue et al., 2015; Justet et al., 2017). Importantly these features were also reflected in the general health check of the animals. To validate the imaging biomarkers, apart from consistent histology patterns, the authors demonstrated that plasma exudation began at day 3 measured by BALF protein content accompanied by neutrophilic invasion which was then followed by leukocytes, macrophages and eosinophils at day 7 according to BALF cytology. Concomitant studies relevant to gene expression patterns revealed differential lung gene expression of pro-inflammatory and pro-fibrotic genes in the bleomycin group while further analysis showed that there was differential expression of such genes between the Low and High bleomycin responders.

A very important issue in preclinical models is the efficient representation of the human disease in the murine model. Under realistic conditions, the lung is exposed through the systemic circulation in a continuous or intermittent fashion, which is in direct contrast with the bolus administration in the trachea that may (1) elicit pronounced acute inflammatory responses, (2) achieve inhomogeneous distribution throughout the lung, and (3) fail to properly simulate irreversible progressive fibrosis as seen in patients. Another significant limitation of the wide application of the model currently is the availability of the imaging modalities but progressively it is reasonable that core facilities using small animal MRI and PET/CT will increase since non-invasive translational approaches are encouraged.

Overall, the study of Mahmnutovic-Persson et al. represents a very well-thought and executed study that provides multilevel information at the whole body, cellular, molecular, and imaging levels with important translational implications. The approach provides a solid foundation for advancement of: (1) preliminary screening protocols for: (i) pulmonary toxicity before sizeable funds are allocated to advance a drug to the clinic (ii) selection of appropriate agents to evaluate in more clinically translatable chronic *in vivo* models, and (2) the rational design of clinical trials aiming to: (i) promptly identify patients that will develop progressive DIILD and (ii) assess established and most importantly experimental therapeutic interventions in that population.

## Author Contributions

SIS and SGZ wrote the manuscript and approved the submitted version.

## Conflict of Interest

The authors declare that the research was conducted in the absence of any commercial or financial relationships that could be construed as a potential conflict of interest.

## Publisher's Note

All claims expressed in this article are solely those of the authors and do not necessarily represent those of their affiliated organizations, or those of the publisher, the editors and the reviewers. Any product that may be evaluated in this article, or claim that may be made by its manufacturer, is not guaranteed or endorsed by the publisher.
